# Aetiology of Ludwig van Beethoven’s hearing impairment: hypotheses over the past 100 years – A systematic review

**DOI:** 10.1007/s00405-020-06467-w

**Published:** 2020-11-23

**Authors:** Jan Peter Thomas, Stefan Dazert, Andreas Prescher, Christiane Voelter

**Affiliations:** 1 Department of Otorhinolaryngology, Head and Neck Surgery, Katholisches Klinikum, Ruhr University Bochum, Bleichstr. 15, 44787 Bochum, Germany; 2 Department of Otorhinolaryngology, Head and Neck Surgery, St.-Johannes-Hospital, Academic Teaching Hospital of the University of Münster, Johannesstr. 9-17, 44137 Dortmund, Germany; 3 Institute of Molecular and Cellular Anatomy, RWTH Aachen University, Wendlingweg 2, 52057 Aachen, Germany

**Keywords:** Beethoven, Deafness, Hearing loss, Aetiology, Cause

## Abstract

**Objectives:**

Even 250 years after Beethoven’s birth, the irrevocable cause of his hearing impairment remains unclear despite multiple publications by different professional groups. This study aimed to analyse the development of the most likely aetiologies during the last 100 years by a systematic review of the relevant medical literature.

**Methods:**

A systematic review of medical literature in PubMed^®^, PubMed Central^®^, and Web of Science^®^ for the period 1920–2020 was conducted. Medical publications between 1920 and 1935 were additionally searched manually by review of reference lists. Studies were eligible when a statement regarding the most likely aetiology of the hearing loss of Beethoven was the aimed objective of the publication.

**Results:**

48 publications were included. The following aetiologies were supposed: otosclerosis (*n* = 10), syphilis (*n* = 9), Paget’s disease (*n* = 6), neural deafness (*n* = 5), immunopathy with inflammatory bowel disease, neural deafness with otosclerosis, sarcoidosis or lead intoxication (*n* = 2), and systemic lupus erythematosus, trauma, labyrinthitis or inner ear disease (*n* = 1). There is an ongoing effort with a mean publication frequency in this topic of 0.48/year. From 1920 to 1970, otolaryngologists were the group with the highest interest in this field (67%), whereas since 1971 most authors have belonged to non-otolaryngologic subspecialities (81%).

**Conclusion:**

Over the past 100 years, otosclerosis and syphilis were predominantly supposed to be the underlying causes. The hypothesis of syphilis—although rejected for a long time—has had a remarkable revival during the past 20 years. Regarding the outcome following therapeutic intervention by cochlear implantation, the differential diagnosis of neural deafness would be relevant today.

## Introduction

The hearing impairment of the composer Ludwig van Beethoven is one of the best-known cases of hearing loss among historical artists. It has attracted great interest over centuries as one might speculate that perfect hearing is essential to be able to create musical masterpieces like Beethoven did. Ludwig van Beethoven represents one of the best examples that the creation of ingenious musical masterpieces does not necessarily require an intact auditory feedback loop [1]. However, analyses of compositions from different periods of his life might reflect the influence of a high-frequency hearing loss at the beginning of his hearing impairment, leading to a diminished usage of high pitches in his compositions at this period of time [2]. Several controversial publications have dealt with the impact of Beethoven’s impaired hearing status onto his compositional work from a musicological point of view in the past years [3, 4].

From the medical point of view, the scientific interest mainly focuses on the aetiology of his hearing impairment which is still the source of ongoing controversial scientific discussions on whether Beethoven might have benefitted from modern concepts of hearing rehabilitation such as cochlear implantation, which is today’s treatment of choice in subjects with severe to profound hearing loss.

Therefore, the aim of this study was (1) to create a systematic review of the medical literature listed in medical databases over the past 100 years on the aetiology of Beethoven’s hearing loss and (2) to critically discuss the pros and cons of the most frequently stated diagnoses.

To evaluate the aetiology of Beethoven’s hearing loss, one mainly has to rely on contemporary primary sources. The medical history can be reconstructed by letters of the composer, his conversational books and documents from his physicians, biographers and other contemporaries. Results of medical examinations are documented in the protocol of the post-mortem examination in 1827 and additionally by a more recent analysis of the bone fragments of his skull in 1985 and 2005 and of his hair in 2000 [5–7].

### Presentation of the hearing loss in the primary literature

Ludwig van Beethoven was born in December in 1770 in Bonn, Germany (the official baptism date was December 17th) and died on March 26th in 1827 at the age of 56 in Vienna, Austria. The first note of a hearing impairment was mentioned by the composer himself in two letters to his friend F. G. Wegeler written on June 29th and November 16th in 1801:

“That malicious demon, however, bad health, has been a stumbling-block in my path; my hearing during the last three years has become gradually worse […].

My ears are buzzing and ringing perpetually, day and night. I can with truth say that my life is very wretched; for nearly 2 years past I have avoided all society, because I find it impossible to say to people, *I am deaf!* In any other profession this might be more tolerable, but in mine such a condition is truly frightful […].

I hear none of the high notes of instruments or singers.[…] I often can scarcely hear a person if speaking low; I can distinguish the tones, but not the words, and yet I feel it intolerable if anyone shouts to me […]”

“The ringing and buzzing in my ears have certainly rather decreased, particularly in the left ear, in which the malady first commenced, but my hearing is not at all improved; in fact I fear that it is become rather worse […]” [8]

Herein, Beethoven gave a detailed description of his hearing impairment which started in the high frequencies with a progressive hearing loss initially affecting the left ear more than the right ear. Besides, he complained about a loss of sound discrimination and permanent tinnitus and showed signs of a positive recruitment.

Also in 1802, Beethoven mentioned his hearing impairment in the “Heiligenstädter Testament”, a letter to his brothers Kaspar Karl und Johann from 1802:

“[…] But you must remember that six years ago I was attacked by an incurable malady […] I was yet obliged early in life to isolate myself, and to pass my existence in solitude. If I at any time resolved to surmount all this, oh! how cruelly was I again repelled by the experience, sadder than ever, of my defective hearing!–and yet I found it impossible to say to others: Speak louder; shout! for I am deaf! […].

Completely isolated, I only enter society when compelled to do so. I must live like an exile.

My intelligent physician recommended me to spare my hearing as much as possible, which was quite in accordance with my present disposition, though sometimes, tempted by my natural inclination for society, I allowed myself to be beguiled into it. But what humiliation when any one beside me heard a flute in the far distance, while I heard nothing, or when others heard a shepherd singing, and I still heard nothing! […].

Ah! how could I possibly quit the world before bringing forth all that I felt it was my vocation to produce? And thus I spared this miserable life–so utterly miserable that any sudden change may reduce me at any moment from my best condition into the worst […].

My brothers Karl and Johann, as soon as I am no more, if Professor Schmidt be still alive, beg him in my name to describe my malady, and to add these pages to the analysis of my disease, that at least, so far as possible, the world may be reconciled to me after my death.[…].

I joyfully hasten to meet death. If he comes before I have had the opportunity of developing all my artistic powers, then, notwithstanding my cruel fate, he will come too early for me, and I should wish for him at a more distant period; but even then I shall be content, for his advent will release me from a state of endless suffering. Come when he may, I shall meet him with courage. Farewell! […] [8]

In 1802, the substantial impact of the progressive hearing loss on his mental state was already described. Social isolation, obvious signs of depression and suicidal tendencies were found. Moreover, Beethoven explicitly asked the medical posterity to figure out the aetiology of his hearing loss in this document.

According to these most cited documents, Beethoven’s hearing loss started in 1796 at the age of 26. In the subsequent years, his hearing thresholds deteriorated so that Beethoven began to use ear trumpets in 1813 which were constructed for him by J. N. Mälzel. Another tool to alleviate the hearing impairment—however also with limited benefits—was a resonating plate which was placed on the piano and reflected the soundwaves [9].

In 1814, the composer Louis Spohr attributed a high-grade hearing loss to Beethoven. However, an oral conservation was still possible in 1815 by speaking into the left ear of the composer [10], while Beethoven seemed to suffer from a more progressive hearing loss on the right ear during the course of the disease. Due to the ongoing progress of the hearing loss, Beethoven started to use conversation books for communication in 1818. In January 1819, the poet P. D. A. Atterboom described Beethoven as deaf as a block, and in May 1824 Beethoven did not even hear the frenetic jubilation at the premiere of his 9th symphony anymore, and had to be turned over by a singer to receive the applause of the audience [10].

The protocol of the autopsy which was performed 1 day after his death on March 27th in 1827 by the assistant of the Institute of Pathology in Vienna J. Wagner reveals some details regarding the ear and the cochlear nerves:

“The external auditory canal appeared to be lined with glistening cutaneous scales; this was especially marked near the concealed tympanic membrane. The Eustachian tube was very thickened and its mucosa rugose and rather narrowed at the bony part. The mastoid process was large and not marked off by any groove; its cells were conspicuous and lined by a highly-vascular mucosa. The whole of the petrous portion of the temporal bone, showing large vessels, was similarly well supplied with blood, especially in the region of the cochlea, the spiral lamina which seemed slightly reddish.”

**Fig. 1:**
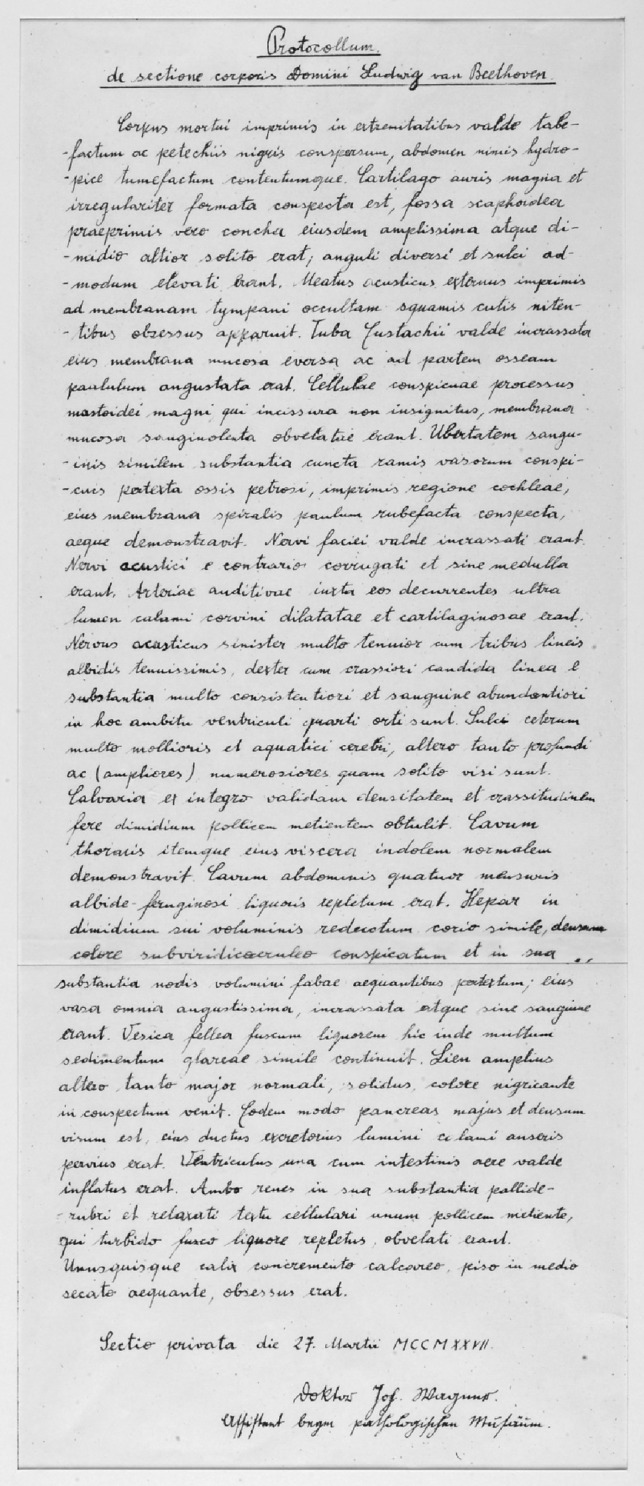
Postmortem protocol of the autopsy of L. v. Beethoven performed by Dr. J. Wagner (reprint with kind permission of the Pathological-Anatomical Collection of the Medical University of Vienna, Austria)

The facial nerves were of considerable thickness; the auditory nerves on the other hand shrunken and without pith; the accompanying auditory arteries were of a calibre of a crow-quill, and of cartilaginous consistency. The left auditory nerve, much the thinner, arose by three very thin, greyish roots; the right by one root, stronger and pale white […]. The vault of the skull showed great tightness throughout and a thickness of about half an inch.” (Fig. 1) [10]

However, there is no description of the middle ear structures available. According to G. Breuning, physician and son of a long-time friend of Beethoven, the temporal bones of the composer were stored in a jar for further examination after autopsy at the Institute of Anatomy at the University of Vienna because the anatomist J. Hyrtl (1810–1894) stated that he had still seen them on the desk of the dissection attendant A. Datter [11]. However, according to further information at a later stage, they have been lost without any hint until today.

Beethoven himself and his physicians associated his hearing loss with his lifelong abdominal problems resulting in diarrhea, impaired digestion, and abdominal colics first mentioned at the age of 25:

“The chief cause of this infirmity proceeds from the state of my digestive organs, which, as you know, were formerly bad enough, but have latterly become much worse, and being constantly afflicted with diarrhea, has brought on extreme weakness. my hearing became worse, and my digestion continued in its former plight.[…] I suffered from most dreadful spasms, and sank back into my former condition. digestive pills four days ago, and a tea for my ears. I certainly do feel better and stronger” [8].

Other diseases temporally associated with the beginning of the hearing loss were an unspecified “dangerous illness [in 1796] which shock hits his hearing tools, from which time his deafness gradually increased” [10] and the description of the physician A. Weißenbach that “he once survived terrible typhoid; from this time on, the deterioration of his nervous system and probably also the embarrassing deterioration of his hearing are dated” [12].

In addition to the life-long abdominal problems, Beethoven also suffered from other diseases. According to several authors who try to place all of Beethoven’s symptoms under one single umbrella, the other diseases might be partially related to his hearing loss. In young adult life, Beethoven suffered from an arthropathy and in 1821 from jaundice. A painful eye condition in 1823 lasting for about 6 months was by some classified as an inflammatory ocular disease. Hematemesis and epistaxis which he suffered from in 1824 and 1825 might have been a prelude to the terminal illness of liver cirrhosis with a chronic pancreatitis. In this context, at the end of 1826/beginning of 1827, Beethoven was affected by extensive ascites and tapped four times before his death in March 1827.

## Material and methods

### Data extraction

For a systematic review of the medical literature of the last 100 years on the aetiology of Beethoven’s hearing impairment, a computer-based search of medical publications was performed following the PRISMA guidelines [13]. Due to the topic of the study, the search process had to be adapted to the particularities of a medical historical theme. Three electronic databases including PubMed^®^, PubMed Central^®^, and Web of Science^®^ were used. The search was carried out from January 1, 1920, to June 30, 2020. The applied search terms in PubMed^®^ and PubMed Central^®^ were “Beethoven” in conjunction with “1920:2020[dp]“ AND “deafness” or “hearing loss”. Search terms in Web of Science were “Beethoven* deafness” or “Beethoven* hearing loss”. After deduplication, two independent reviewers separately screened for eligibility and in case of incongruency a third reviewer provided consent. Titles and, if available, abstracts were screened for inclusion or exclusion.

As a first step, publications which did not deal with the medical history of the composer Beethoven (e.g., Tmc1 Beethoven mouse model) were excluded; as a second step, publications written in other languages than English, German, Spanish, Italian and French were discarded; and as a third step, publications which did not deal with the aetiology of Beethoven’s hearing loss were excluded. Then, the full-text versions of all publications that passed the screening were thoroughly evaluated. As a fourth step, further publications which do not deal with the aetiology of Beethoven’s hearing loss were discarded. As a fifth step, publications which did not give a statement regarding the underlying cause of the hearing loss were excluded. Studies were considered eligible if they comprised the description of a single favoured aetiology or if a clear exclusion of a cause was at least one of the study aims.

Currently Pubmed^®^ covers only medical literature at the earliest from 1936 to the present day. Therefore, to get complete access to all publications published in medical journals over the past 100 years, medical publications published between 1920 and 1935 were additionally searched manually by review of all reference lists of the above-mentioned eligible papers.

## Results

**Fig. 2:**
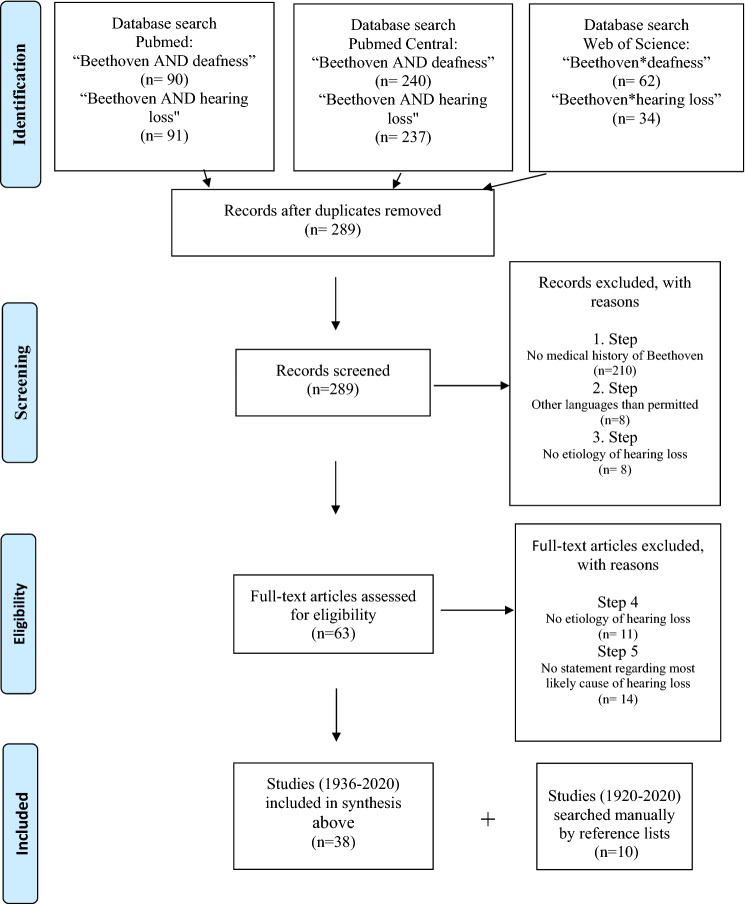
Flowchart of literature retrieval

The results of the literature retrieval process regarding the medical literature from 1920 up to now is illustrated in the flow diagram depicted in Fig. 2. After careful evaluation of the medical literature between 1936 and 2020, 38 medical publications on the cause of Beethoven’s deafness met the eligibility criteria for inclusion in this critical review. Retrieval for eligible literature from 1920 to 1935 revealed a further ten publications. Hereby, 30 original/review articles, 13 historical notes, 3 letters to the editor, 1 conference paper, and 1 editorial note were included. The overall 48 eligible publications are chronologically listed in Table 1 including the type of article and the medical speciality of the first author. If there was more than one publication from the same author group favouring the same most likely aetiology, the results are listed in the same line to prevent distortion of the presented results.

**Table 1:** Chronological order of medical publications (1920–2020) dealing with the most likely cause of the hearing loss of L

Year of publication	Authors	Type of article	Medical specialty of the author	Most likely etiology
2020	Brotto D et al.	O/R	ORL	Chronic lead poisoning
2018	Pedro MKF et al.	HN	Neurology	Syphilis
2018	McGill IK and Agius MA	CF	Psychiatry	Tertiary syphilis
2017	Oiseth SJ	O/R	Pathology	Paget‘s disease
2013	Stevens MH et al.	O/R	ORL	Chronic lead exposure
2008	Domic D and Payá E	HN	Infectiology	Syphilis
2005	Karmody CS and Bachor ES	O/R	ORL	Immunopathy with IBD
2001/00	Huxtable RJ	HN/O/R	Pharmacology	Neural deafness nos
2001	Wolf P	HN	Pathology	Paget‘s disease
2000	Donnenberg MS et al.	O/R	Infectiology	Syphilis
1996	Kubba AK and Young M	O/R	Gastroenterology	Mixture nerve deafness and otosclerosis
1994/92/90	Palferman TG	O/R/LE/HN	Rheumatology	Sarcoidosis
1994	Drake ME	HN	Neurology	Sarcoidosis
1992	Wintersgill P	HN	Medicine	Otosclerosis
1990	Shearer PD	O/R	Pediatrics	Cochlear otosclerosis
1988/1995	Davies PJ	O/R/O/R	Gastroenterology	Immunopathy with IBD
1986	Jesserer H and Bankl H	O/R	Pathology	Exclusion of Paget’s disease
1978	Landsberger M	O/R	Pediatrics	Otosclerosis
1974	Sellars SL	HN	ORL	Otosclerosis, ostitis deformans
1971/71	Naiken VS	O/R/LE	Pathology	Paget‘s disease
1971/70	Larkin E	EN/HN	Neurology	Connective tissue disorder, SLE
1970	Kerner D	O/R	Medicine	Syphilis
1970	Miller JW	LE	Unknown	Trauma of cochlea and acoustic nerve
1970	Stevens KM and Hemenway WG	HN	ORL	Cochlear otosclerosis
1965	Coude Jahn F	O/R	ORL	Otosclerosis
1965	Asherton N	HN	ORL	Pagets disease
1963	Sellers LM	O/R	ORL	Neurovascular hearing loss
1960	Cawthorne T	O/R	ORL	Otosclerosis
1958	McCabe BF	O/R	ORL	Syphilis
1937	Squires PC	O/R	Psychiatry	Acquired syphilis
1937	Magenau C	O/R	ORL	Inner ear disease nos
1936	Vorhees IW	O/R	ORL	Syphilis
1935	Frank I	O/R	ORL	Otosclerosis
1934	J I S	HN	Unknown	Otosclerosis and nerve deafness
1932	Tremble E	O/R	ORL	Nerve affection by gastrointestinal disturbances
1930	Sorsby M	O/R	ORL	Exclusion of syphilis
1928	Schultze F	O/R	Neurology	Nerval deafness not necessarily caused by syphilis
1928	Marage M	O/R	ORL	Labyrinthitis due to cerebral or intestinal cause
1927	Jacobsohn J	HN	Neurology	Syphilis
1927	Neumann H	O/R	ORL	Paget’s disease or otosclerosis
1921	Gradenigo G	O/R	ORL	Otosclerosis
1920	Schweisheimer W	O/R	Unknown	Neuritis acoustica

Beethoven (*O/R* Original or Review Article, *HN* historical note, *CF* conference paper, *EN* editorial note, *LE* letter to the editor, *IBD* inflammatory bowel disease, *nos* not otherwise specified)

**Fig. 3:**
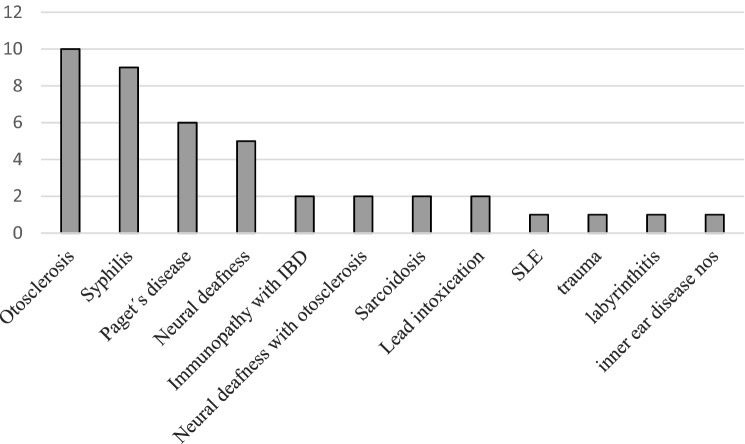
Frequencies of the supposed most likely causes of the hearing loss of Beethoven according to the number of author groups in the medical literature 1920–2020 (*SLE* systemic lupus erythematosus, *IBD* inflammatory bowel disease, *nos* not otherwise specified)

The number of the most likely supposed cause of Beethoven’s hearing loss is shown in Fig. 3 according to each author group. To prevent distortion of the results, the most likely diagnoses were counted only once for every author if the author had more than one publication. The four most frequent diagnoses were otosclerosis (*n* = 10), syphilis (*n* = 9), Paget’s disease (*n* = 6) and neural deafness not otherwise specified (*n* = 5).

## Discussion

Even 250 years after Ludwig van Beethoven’s birth, medics still deal with the cause of his deafness which is still obscure. We are presenting the first systematic review of medical publications on the most likely cause of Ludwig van Beethoven’s deafness. Over the past 100 years, there was a mean release frequency of about one new publication supposing an underlying aetiology of his hearing loss in the medical literature in every 2.1 years with a quite continuous publication activity except for the period during and after the Second World War.

Regarding the subspecialities of the authors, there is an obvious change in the publications on this topic over the past 100 years. Whereas from 1920 to 1970, otolaryngologists represented the dominating discipline with 14 of 21 publications, in the past 50 years only 4 of 21 author groups originate from otolaryngology. So, experts from different disciplines like pharmacologists, pathologists, infectiologists, gastroenterologists, rheumatologists, neurologists, psychiatrics and even paediatricians might be increasingly interested in this topic.

In the literature, several differential diagnoses regarding the aetiology of Beethoven’s hearing loss have been controversially discussed. According to the number of publications, the leading cause supposed to explain Beethoven’s hearing loss is otosclerosis which was already favoured in the medical literature between 1921 and 1935 by otolaryngologists [14, 15] and later in the 1970s and 1990s by a wide range of authors of different medical specialities [16, 17]. Arguments supporting this diagnosis have been the age when Beethoven first experienced symptoms of hearing loss in his mid-twenties and the progressive decline of his hearing affecting both ears. In addition, some authors diagnosed conductive hearing loss in Beethoven based on a wooden drumstick that Beethoven applied to the piano and held on the other side between his teeth which is comparable to a bone conduction hearing aid. However, the only description of such a drumstick was done by the French physician J.A.A. Rattel, who was not a contemporary of Beethoven. Thus, the mentioned sort of conductive hearing aid might be classified more legendary than real nowadays [18]. In contrast, the medical history of Beethoven with a hearing impairment initially involving the high frequencies, a loss of discrimination, and signs of a positive recruitment is not typical in early stages of a fenestral otosclerosis, but point more to a cochlear hearing impairment. So, combined or cochlear otosclerosis might be a justifiable alternative differential diagnosis as mentioned by Shearer [17]. In the postmortem protocol of Beethoven, no otosclerotic involvement of the oval window was reported, although this might easily have been visible even without a microscope according to Sorsby who excluded a combined otosclerosis [19]. The remaining pure cochlear otosclerosis is defined as a focus of otosclerosis located in the otic capsule involving the cochlear endosteum, causing sensorineural hearing loss without any stapes fixation. However, the prevalence of pure cochlear otosclerosis might be low even if the current data situation is still unclear. Whereas clinical–radiological studies report patients with a pure cochlear sensorineural type of otosclerosis [20], otopathologic studies indicate that this type of pathology is very rare with a prevalence of only 1% of all patients with otosclerosis [21]. According to these data, Beethoven would have suffered from a very rare sub-entity of otosclerosis if cochlear otosclerosis was the underlying cause of his hearing impairment.

Syphilis is the aetiology second most accused of being the cause of Beethoven’s hearing loss. In the nineteenth century, syphilis was a very common venereal infectious disease which was considered the cause of Beethoven’s deafness by several non-medical authors and the composer’s biographers. The non-physician G. Grove was the first who mentioned this in the first edition of his musicology dictionary “Grove’s dictionary of Music and Musicians” in 1879 by the entry “the whole of these [postmortem] appearances are most probably the result of syphilitic affections at an early period of his life”. This statement had been initiated by third-hand information of the biographer A. W. Thayer who assumed Beethoven had syphilis based on two prescriptions for Beethoven by his physician Dr. Bertolini in 1820 for medication containing mercury [22]. Even in the 1920s and 1930s, some authors still favoured the hypothesis of tertiary syphilis as the cause of his hearing loss [23]. However, in 1930 the otolaryngologist Sorsby published arguments against syphilis as the cause of Beethoven’s hearing loss. He stated that no sudden onset or sudden total deafness or vertigo or nystagmus as commonly seen in syphilis patients had been described in Beethoven’s medical history [19]. So, in the following decades syphilis was only sporadically mentioned [24], and Shearer mentioned even in 1990 that McCabe was still the only modern otologist who defended the aetiology of syphilis [17, 24]. Even if a tertiary syphilis might explain the mentioned atrophy of the cochlear nerve due to meningeal irritation, and even if an uveitis as an inflammatory ocular disease might be part of a neurosyphilis complex, there were no other signs of advanced syphilis such as headache, hemiplegia, dysphasia and seizures and no autoptic evidence as gumma and tabes dorsalis. Also, the early beginning of the hearing loss at the age of 26 years is unlikely in tertiary neurosyphilis, and the pace of progression of the hearing impairment is different from the pace of progression in meningovascular syphilis patients [25]. In addition to the missing typical anamnestic and autoptic findings regarding the signs of syphilis in Beethoven, a more recent examination of a ringlet of the composer in 2000 demonstrated that there was no detectable mercury in Beethoven’s hair which makes syphilis therapy with mercury unlikely [26]. In opposition to these arguments against syphilis as the cause of Beethoven’s hearing impairment, the syphilis diagnosis has gained interest again in the medical literature of the twenty-first century by up to now four authors with medical specialization in infectiology, neurology, and psychiatry favouring this aetiology anew [27–30]. Overall, syphilis is still one of the aetiologies which has most often been accused of being the cause of Beethoven’s hearing loss by medical publishers over the past 100 years. However, in the literature published by otolaryngologists, this differential diagnosis has not been favoured any more during the last 50 years even if nowadays the diagnosis of otosyphilis still needs to be considered in the differential diagnostic mind of the clinical otologists as recently mentioned by Theeuwen [31].

Besides otosclerosis, another bone-remodelling disease with fixation of the stapes, the Paget´s disease, was accused of being the cause of Beethoven’s deafness in perennial publications from 1965 up to these days [32, 33]. About 50% of patients with Paget’s disease are known to suffer from hearing loss which can be caused by multiple mechanisms including, e.g., a fixation of the stapes footplate, a diminished mineral density of the cochlear capsule with microfractures and a damage of the cochlear nerve by a bony overgrowth and compression in tiny foramina before and within the internal auditory canal [33]. The authors supporting this hypothesis based their argumentation on the report that the dense skull was twice as thick as normal and on the documentation of a narrowed bony part of the Eustachian tube in the autopsy protocol of Beethoven as well as on Beethoven’s facial characteristics such as his big head and jaw. Furthermore, an atrophy of the cochlear nerve which was mentioned in the autopsy protocol and Beethoven’s statement that he completely lost his ability to hear after a fall in 1815 [8] were appraised as secondary clues for an underlying Paget´s disease. In addition, an increased blood supply and hyperplasia of the vessels of the temporal bones as mentioned in the autopsy protocol of Beethoven can also be seen in patients with Paget’s disease. However, only 3% of patients with Paget’s disease present symptoms under the age of 30 years [34], and in most cases hearing impairment initially starts with either a mixed or a conductive hearing loss affecting mostly the lower frequencies in contrast to Beethoven who presented with a hearing loss in the high frequencies. In addition, in 1986 the pathologists Jesserer and Bankl examined parietal bone fragments of the skull of Beethoven without finding any macropathological or radiological signs of Paget’s disease. Bilateral involvement of the temporal bones by osteodystrophic changes in the mid-twenties without any inclusion of the adjacent parietal parts up to the fifth decade of life have to be considered as quite unlikely [5].

From 1920 up to the turn of the millennium, neural deafness was periodically accused of being the underlying cause of Beethoven’s hearing impairment [35, 36]. This assumption was mainly based on the fact that the atrophy of the cochlear nerves which was described could not entirely be explained by any other aetiology. However, a convincing pathophysiologic cause was not answered by these publications [35, 37]. Furthermore, the anamnestic cues of a positive recruitment by Beethoven himself are not compatible with the hypothesis of a neural origin, but rather point more towards a cochlear hearing impairment.

Beethoven himself assumed a relationship between his lifelong gastrointestinal problems and his hearing impairment. This is also reflected throughout the medical literature during the last 100 years. While assumptions about a toxic labyrinthitis or an unspecified cochlear nerve disorder caused by intestinal problems which were popular in the 1920s and 1930s [37, 38] are no longer tenable today, the assumption of an inflammatory bowel disease (IBD) associated with an immunopathy of the inner ear was first expressed by Davies in 1988 and elaborated later by Karmody in 2005 [40]. This goes in line with the first description of an autoimmune sensorineural hearing loss by McCabe in 1979 [39–41]. Several authors attributed Beethoven’s intestinal disorder to ulcerative colitis [42] or more rarely to Crohn’s disease [43] which are subsets of IBD. Especially in ulcerative colitis, an autoimmune-mediated sensorineural hearing loss might become an extraintestinal manifestation of an IBD [44] and an underlying ulcerative colitis also might explain other maladies of Beethoven such as joint pains, facial rash, and uveitis [40]. However, up to now research regarding extraintestinal otologic manifestations of ulcerative colitis is rare and the prevalence of a profound hearing loss as a consequence of an IBD has not yet been clarified. One argument against the hypothesis of an autoimmune-mediated sensorineural hearing loss is the period of appearance of Beethoven’s hearing impairment; an autoimmune-mediated hearing loss usually occurs during weeks or months but not years as it was the case with Beethoven [45]. In addition, according to the primary literature there was no history of rectal bleeding during his lifetime and the autopsy protocol revealed an intestinal tract filled with air and without adhesions, strictures, or perforations. This strongly argues against the hypothesis that Beethoven suffered from IBD at all [46].

In the 1990s, Drake and Palverman favoured independently of each other sarcoidosis as a diagnosis, as sarcoidosis encompasses most of the different diseases that Beethoven had. The multisystemic involvement of sarcoidosis can cause chronic active hepatitis followed by cirrhosis, inflammatory bowel disease, post-dysenteric seronegative arthropathy, hypercalcemia, and nephrolithiasis [47]. In 5% of subjects with sarcoidosis, the nervous system is involved. Neurosarcoidosis might explain Beethoven’s deafness [48]. But although bilateral auditory nerve deafness has been reported in sarcoidosis, this is associated with a thickening but not with an atrophy of the cochlear nerves as it was described in Beethoven’s autopsy protocol. In addition, neurosarcoidosis usually encompasses a clinical involvement of the facial nerve which was not found in Beethoven. Not least, no gross pathology of lung and heart was mentioned in his autopsy protocol.

It lasted up to 2013, that lead intoxication was mentioned in the medical literature as a potential cause of Beethoven’s hearing loss. Evaluations of Beethoven’s hair and parietal skull bones in the first decade of this century revealed substantially elevated lead levels indicating chronic lead exposure which might have caused lead poisoning [6, 7]. Whereas subacute lead poisoning notoriously induces a motor neurone disorder, long-term lead exposure can result in a slowly progressive high-frequency hearing loss [49] probably caused by axonal degeneration of the cochlear nerve. Stevens explained a chronic lead exposure of Beethoven by his long-lasting consumption of wine, which might have started at the age of 17 after the death of his mother. At that time, lead was added to improve the flavour of cheaper wines like the Hungarian ones preferred by Beethoven [45]. Other sequelae of chronic lead intoxication such as abdominal colic, hepatotoxicity, and kidney damage as reported by Beethoven support this hypothesis. Recently, Brotto underlined this hypothesis with regard to Beethoven’s autoptic findings of wrinkled cochlear nerves that lacked pith without a medulla [50]. However, up to now the scientific data regarding the prevalence and extent of hearing loss induced by chronic lead exposure is still rare. Therefore, it remains difficult to clearly associate Beethoven’s hearing impairment with a chronic exposure of lead.

## Conclusion

Beethoven’s explicit wish to clarify the cause of his deafness after his death has been fulfilled in numerous medical publications. Based on primary historical sources including the autopsy report and more recent investigations of probes of his skull and hair, several different hypotheses on the aetiology of the hearing impairment of Beethoven have been postulated.

Otosclerosis and syphilis have been the predominant supposed causes in the medical literature of the past 100 years, whereby the long-rejected hypothesis of syphilis has shown a surprising and probably questionable revival during the last 20 years. In addition to numerous other causes, the hypothesis of a chronic lead intoxication has recently been developed in the medical literature over the past decade.

To date, no single aetiology exists which might on its own completely explain Ludwig van Beethoven’s hearing impairment without a pertinent argument against this hypothesis. So, it is most likely that the question regarding the irrevocable cause of his deafness will probably remain unanswered forever.

Whereas during his lifetime only insufficient hearing rehabilitation via hearing aids in the form of ear trumpets and a resonating plate was available, it is supposed that Beethoven would now probably benefit from an electrical stimulation by a cochlear implant. In this context, it has to be mentioned that the composer anticipated the physical fundamentals of this therapeutic option already at the beginning of his hearing loss when he wrote in 1801: “I hear wonders of galvanism; what do you say to it? A physician told me that he knew a deaf and dumb child whose hearing was restored by it, and likewise a man who had been deaf for seven years, and recovered his hearing” [8]. To predict the outcome of such a treatment, the irrevocable identification of the aetiology of his hearing loss—especially regarding the possible neural pathophysiologic cause—would be of therapeutic importance.
